# Fueling the rush – it’s all about caffeine

**DOI:** 10.1080/15502783.2025.2550145

**Published:** 2025-08-25

**Authors:** Lia Jiannine, Catherine Holen, Tobin Silver, Jose Antonio

**Affiliations:** Nova Southeastern University, Department of Health and Human Performance, Davie, FL, USA

**Keywords:** Energy drink, attention, memory, reaction time

## Abstract

**Background:**

The primary aim of this study was to assess the effectiveness of an energy drink vs. a caffeine-matched control on measures of sustained attention.

**Methods:**

Using a randomized, double-blind design (*n* = 107), subjects were assigned to an energy drink (C4 – Nutrabolt®) or a caffeine-matched control (200 mg caffeine). The following assessments were conducted before and approximately 45 minutes after consuming an energy drink or caffeine-matched control: psychomotor vigilance test (PVT), Memtrax (memory), handgrip strength, and wall-sit endurance.

**Results:**

Both the energy drink and the caffeine-matched control improved pre vs. post for the PVT; however, there were no differences in the delta score. The energy drink group had a significant decrease in the Memtrax assessment (% correct) whereas reaction time increased with no change in the control; however, there were no differences in the delta score between groups for either the % correct score or reaction time. There were no significant differences in the handgrip or wall-sit assessments for either group.

**Conclusions:**

Consuming 200 mg of caffeine, whether it is part of an energy drink or is a stand-alone ingredient, induces significant improvements in sustained attention and alertness (i.e. reaction time assessed by the PVT). However, there were no differences in memory, handgrip strength, or wall-sit endurance.

**Table 1.** Physical characteristics.

	Treatment *N* = 61 (18 male)	Control *N* = 56 (19 male)	P value
Age years	21 ± 3	20 ± 1	.4083
Height cm	169 ± 11	171 ± 10	.4155
Body mass kg	69.2 ± 15.8	75 ± 29	.2021
Body fat %	23 ± 9	23 ± 9	.7457
Aerobic exercise/week minutes	239 ± 268	198 ± 244	.4024
Resistance exercise/week minutes	186 ± 161	198 ± 151	.6917
Other exercise/week minutes	78 ± 200	111 ± 234	.4175

Data is the mean and standard deviation (SD). There were no significant differences between the groups.

**Table 2.** Performance metrics.

	Treatment	Control	*p* value delta
PVT pre (msec)	371 ± 102	380 ± 101	
PVT post (msec)	356 ± 97*	354 ± 101*	
Delta	−15 ± 59	−26 ± 74	0.3426
Memtrax % correct pre	95 ± 5	91 ± 11	
Memtrax % correct post	92 ± 6*	93 ± 5	
Delta	−3 ± 5	2 ± 11	0.1596
Memtrax RT pre (sec)	0.77 ± 0.14	0.78 ± 0.18	
Memtrax RT post (sec)	0.79 ± 0.13*	0.78 ± 0.17	
Delta	0.02 ± 0.10	0.00 ± 0.12	0.1596
Handgrip pre (kg)	38 ± 13	38 ± 13	
Handgrip post (kg)	39 ± 13	38 ± 13	
Delta	1 ± 4	0.0 ± 0.1	0.5485
Wall sit (sec)	111 ± 78	111 ± 80	

Data is the mean and SD. *Significantly different than the pretest. PVT treatment *p* = 0.0344, PVT control *p* = 0.0070, Memtrax % correct treatment *p* < 0.0001, Memtrax reaction time treatment *p* = 0.307. There were no significant differences for any of the other assessments (pre vs post), the wall sit, and the delta scores between groups. Legend: kg – kilogram, msec – milliseconds, sec – seconds.

